# The Role of the APC/C and Its Coactivators Cdh1 and Cdc20 in Cancer Development and Therapy

**DOI:** 10.3389/fgene.2022.941565

**Published:** 2022-06-27

**Authors:** Christine Greil, Monika Engelhardt, Ralph Wäsch

**Affiliations:** Department of Hematology, Oncology and Stem Cell Transplantation, Faculty of Medicine, Medical Center-University of Freiburg, Freiburg, Germany

**Keywords:** mitotic exit, APC/C, Cdh1, mitotic slippage, spindle assembly checkpoint, antimitotic therapy

## Abstract

To sustain genomic stability by correct DNA replication and mitosis, cell cycle progression is tightly controlled by the cyclic activity of cyclin-dependent kinases, their binding to cyclins in the respective phase and the regulation of cyclin levels by ubiquitin-dependent proteolysis. The spindle assembly checkpoint plays an important role at the metaphase-anaphase transition to ensure a correct separation of sister chromatids before cytokinesis and to initiate mitotic exit, as an incorrect chromosome distribution may lead to genetically unstable cells and tumorigenesis. The ubiquitin ligase anaphase-promoting complex or cyclosome (APC/C) is essential for these processes by mediating the proteasomal destruction of cyclins and other important cell cycle regulators. To this end, it interacts with the two regulatory subunits Cdh1 and Cdc20. Both play a role in tumorigenesis with Cdh1 being a tumor suppressor and Cdc20 an oncogene. In this review, we summarize the current knowledge about the APC/C-regulators Cdh1 and Cdc20 in tumorigenesis and potential targeted therapeutic approaches.

## The Role of Cdh1 and Cdc20 in Cell Cycle Regulation

**FIGURE 1:**
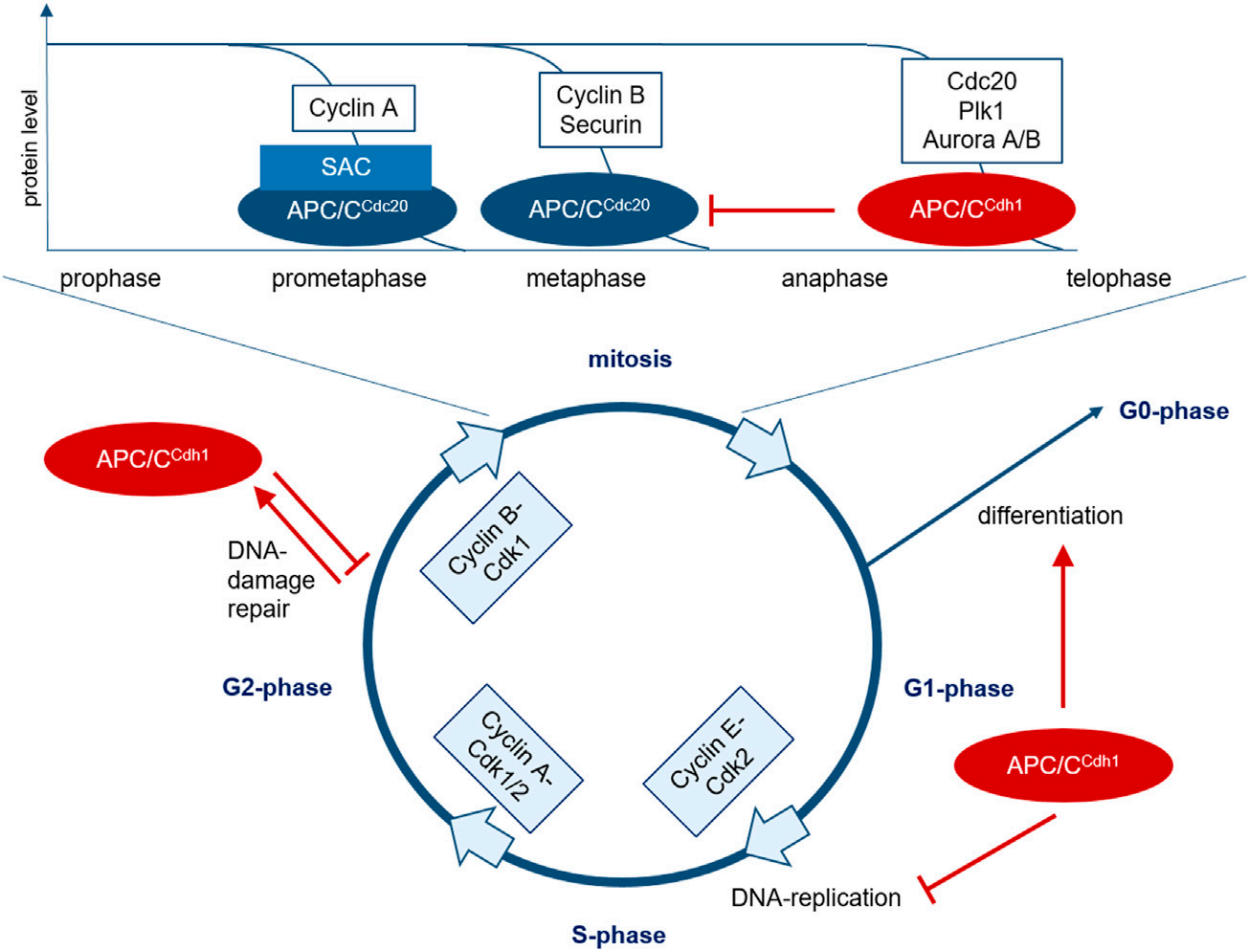
The role of APC/C in cell cycle regulation. The transition between the cell cycle phases is regulated by the cyclic activity of cyclin-Cdk complexes. In mitotic entry, cyclin B-Cdk1 plays a crucial role. In early prometaphase, chromosomes bind to the mitotic spindle and improperly connected kinetochores lead to SAC-activation. At first, this partially inhibits substrate recruitment of APC/C activated *via* Cdc20, but various prometaphase proteins such as cyclin A can be further marked for degradation by SAC-APC/C^Cdc20^. After correct attachment of all chromosomes to the mitotic spindle, the SAC is inactivated, leading to degradation of additional proteins such as cyclin B and securin *via* APC/C^Cdc20^ in metaphase, mediating chromosome segregation and initiating mitotic exit. Cyclin B-degradation leads to Cdk1 inhibition, resulting in dephosphorylation and activation of Cdh1. APC/C^Cdh1^ then initiates the degradation of various proteins during ana- and telophase such as Cdc20, Plk1, and Aurora A/B. At the end of mitosis, APC/C^Cdh1^ inactivates APC/C^Cdc20^ and regulates anaphase spindle dynamics and cytokinesis. APC/C^Cdh1^ is thus activated from late mitosis and controls the decision between proliferation and differentiation in G1-phase. In G2-phase, APC/C^Cdh1^ can be activated in response to DNA-damage to prevent mitotic entry and allow DNA-repair.SAC, spindle assembly checkpoint; APC/C, anaphase promoting complex; Plk, Polo-like Kinase; Cdk, cyclin-dependent kinase.

To ensure a correct DNA-duplication and distribution of chromosomes to the daughter cells, the cell cycle is controlled by a complex interaction of different intra- and extracellular factors with proliferation-promoting or -inhibiting effects. The correct sequence of cell cycle phases is regulated by the cyclic activity of cyclin-dependent kinases (Cdk) and their binding to regulatory cyclins (Figure 1) (Evans et al., 1983; King et al., 1996; Miller and Cross, 2001). Before forming of the pre-replication complex (pre-RC) at the origin of replication during the initiation step of DNA replication, the cyclin activity has to be low in G1-phase. DNA replication is initiated with increasing cyclin activity at the G1/2-transition. The high cyclin activity prevents a repeated formation of the pre-RC to ensure only one replication round per cell cycle. Finally, a decreasing cyclin activity allows mitotic exit (King et al., 1996).

The regulation of cyclin activity and thus proceeding in the cell cycle is regulated *via* synthesis and degradation of these proteins, phosphorylation, Cdk-inhibition, and the localization of the enzyme complexes within in the cell. The ubiquitin-dependent proteolysis of cyclins and other regulatory proteins by the proteasome plays a key role in this context (Hershko and Ciechanover, 1998).

There are two important ubiquitin ligases: the Skp1-, Cullin-, and F-box-protein containing-complex (SCF-complex) regulating entry into S-phase *via* degradation of Cdk-inhibitors in G1-phase, and the anaphase-promoting E3 ubiquitin ligase complex or cyclosome (APC/C) that mediates the separation of sister chromatids and mitotic exit by the degradation of regulatory proteins like securin and cyclin B (Harper et al., 2002; Peters, 2006).

Cell cycle checkpoints are control mechanisms to ensure its proper progression. The spindle assembly checkpoint (SAC) plays an important role at the metaphase-anaphase transition and prevents the separation of chromatids until each chromosome is correctly attached to the mitotic spindle to avoid chromosome missegregation (Figure 1). If a cell is not able to resolve an error detected at this checkpoint it initiates programmed cell death (apoptosis). A dysfunctional checkpoint can lead to aneuploidy and tumorigenesis (Greil et al., 2016; Zhang et al., 2014). Several studies have proven the particular significance of the SAC for genetic stability (Scully, 2010; Greil et al., 2016; Lawrence et al., 2015).

An accurate connection of the kinetochores to the microtubules of the mitotic spindle ensures the correct separation of the sister chromatids (Foley and Kapoor, 2013). This interaction is monitored by the SAC to prevent a premature separation and thus chromosomal instability (Musacchio and Salmon, 2007; Musacchio, 2015). Single unattached kinetochores keep the SAC active and mediate the assembly of the mitotic checkpoint complex (MCC), that inhibits the APC/C and thereby stabilizes its substrates and prevents chromosome separation and mitotic exit. The MCC is composed of Mad2, BubR1, Bub3, and the APC/C-co-activator Cdc20 (Sudakin et al., 2001). The inhibitory signal from unattached kinetochores leads to a conformational change of Mad2 and subsequently Cdc20 that thereby is able to bind BubR1 and Bub3 (Skinner et al., 2008; Han et al., 2013), can no longer activate the APC/C and thus inhibits anaphase-entry (Peters, 2006).

A correct arrangement of all chromosomes with bipolar binding to the mitotic spindle in the metaphase plate leads to SAC-silencing and facilitates mitotic exit when Cdc20 is released after MCC-degradation and can activate the APC/C (Zhang et al., 2007). Thus, the APC/C plays a crucial role in the regulation of the M/G1-transition. It consists of 13 subunits and its activity is regulated by the two co-activators Cdc20 and Cdh1 recruiting specific substrates. Their interaction is regulated by phosphorylation and temporally related to the M- and G1-phase (Morgan, 1999; Harper et al., 2002; Peters, 2006; Sullivan and Morgan, 2007; Pines, 2011; Greil et al., 2016). Major substrates of the APC/C are the mitotic cyclins A and B, mitotic kinases such as Aurora A and B and Plk1, proteins involved in chromosome separation as securin and replication proteins like Cdc6. The two co-activators Cdc20 and Cdh1 themselves are degraded APC/C-mediated (Figure 1) (Peters, 2006).

APC/C^Cdc20^ mediates separation of sister chromatids by ubiquitin-mediated degradation of securin. Moreover, it initiates cyclin B-degradation and thereby leads to Cdk1-inactivation (Wäsch and Cross, 2002; Peters, 2006; Musacchio and Salmon, 2007; Schnerch et al., 2012a; Greil et al., 2016; Schrock et al., 2020). After Cdk1-inactivation, the second regulator Cdh1 is able to bind and activate the APC/C. It is active during late mitosis and early G1-phase and controls different cell cycle-regulators to ensure a stable G1-phase after mitotic exit and thereby optimal conditions prior to DNA-replication in the following S-phase and progression in the cell cycle towards either differentiation or division (Figure 1) (Wäsch and Cross, 2002; Qiao et al., 2010; Wäsch et al., 2010). Moreover, APC/C^Cdh1^ is activated during DNA-damage response in G2-phase to prevent mitotic entry and initiate DNA-repair processes (Figure 1) (Sudo et al., 2001; Bassermann et al., 2008).

## The Role of Cdc20 and Cdh1 in Tumorigenesis

The strict regulation of cell growth and division within the cell cycle ensures that each daughter cell receives a complete set of chromosomes. An incorrect chromosomal separation leads to aneuploidy and thus possibly to the loss of tumor suppressor genes or the overexpression of oncogenes (Jallepalli and Lengauer, 2001). Additionally, an inaccurate DNA-replication can lead to mutations with subsequent loss of function of tumor suppressors (Rouse and Jackson, 2002). Mutations in different cell cycle regulators can consecutively result in various mutations leading to genomic instability, the main feature of malignant cells (Negrini et al., 2010). The APC/C-co-activator Cdh1 is considered a potential tumor suppressor (Wang et al., 2015) and it is downregulated in different tumor entities (Engelbert et al., 2008). Heterozygous Cdh1-knockdown (kd) mice have been shown to develop tumors more frequently (García-Higuera et al., 2008). Loss of Cdh1 leads to inefficient proliferation, the accumulation of chromosomal aberrations (Wheeler et al., 2008), elevated sensitivity to DNA-damage (Ishizawa et al., 2011; de Boer et al., 2016) and development of various tumor entities (García-Higuera et al., 2008). After Cdh1-downregulation different APC/C^Cdh1^–substrates such as cyclin A and B, Aurora A and Plk1 can accumulate. Persistent residual levels of the mitotic cyclins A and B at mitotic exit lead to a disturbed DNA-replication (Diffley, 2004), a delayed subsequent mitotic entry of cells with incompletely replicated DNA, may cause disturbed mitosis, stabilization of p53/p21 and finally genomic instability (Engelbert et al., 2008). However, it is not fully understood how Cdh1 inactivation leads to genomic instability (Wäsch and Engelbert, 2005; García-Higuera et al., 2008). The stabilization of mitotic cyclins in G1-phase may lead to premature and prolonged S-phase (Diffley, 2004; Greil et al., 2016; Choudhury et al., 2016). In the following mitosis, the chromosomes that are defective or not completely replicated in the disturbed S-phase lead to further aberrations and ultimately to the malignant transformation of the cell. By stabilization of the mitotic cyclins A and B in the nucleus, Cdh1-kd subsequently leads to a reduced accumulation and chromatin binding of the pre-RC-component MCM4 (Greil et al., 2016). MCM4-accumulation is not restored after additional inhibition of Cdk1, thus, other kinases may also play a role in this context (Wheeler et al., 2008; Greil et al., 2016). Cdh1-kd may lead to a prolonged S-phase because after reduced formation of pre-RC complexes, DNA-replication starts from fewer replication origins in G1-phase (Ayuda-Duran et al., 2014; Greil et al., 2016). Stabilization of cyclin A and B with persistent cyclin A and B-dependent Cdk1-activity and cyclin A and E-dependent Cdk2-activation in G1-phase after stabilization of the SCF-component Skp2 and degradation of the Cdk-inhibitors p27 and p21 may contribute to premature S-phase entry after Cdh1-kd (Bashir et al., 2004; Wäsch and Engelbert, 2005; Yuan et al., 2014). Incomplete DNA-replication after Cdh1-kd can cause double-strand breaks during chromosome condensation and segregation (Greil et al., 2016). As a consequence of this disturbed replication and defective chromosome segregation, mitotic aberrations occur leading to anaphase bridges, micronuclei, impaired cytokinesis, and polyploid cells (Neelsen et al., 2013; Greil et al., 2016). Tetraploid cells formed by re-fusion of daughter cells after insufficient division show supernumerary centrosomes that can either lead to multipolar mitoses or, after clustering to ensure bipolarity, to merotelic chromosome junctions and lagging chromosomes (Ganem et al., 2009; Greil et al., 2016). The APC/C plays a role in this centrosomal clustering and Cdh1-kd can lead to multipolar mitotic spindles by stabilizing the Eg5 motor protein (Drosopoulos et al., 2014). These disturbed processes result in either mitotic catastrophe and apoptosis or accumulation of chromosomal aberrations and tumorigenesis. Downstream of Cdh1-kd, stabilization of topoisomerase 2α has also been described, probably contributing to genomic instability (Eguren et al., 2014). However, the mechanism leading to genomic instability is currently not fully understood (Tavormina et al., 2002). Despite increasing genomic instability, Cdh1-deficient cells can survive and malignant transformation occurs only after a longer latency period and additional mutations (García-Higuera et al., 2008; Skaar and Pagano, 2008).

Interestingly, Cdh1 overexpression may also promote tumorigenesis: High levels of Cdh1 lead to a prolonged G1-phase, delayed entry in S-phase and increased degradation of geminin (Sørensen et al., 2000). Thereby, the pre-RC component Cdt1 is no longer inhibited, which may lead to repeated DNA replication rounds and thus to aneuploidy (Wohlschlegel et al., 2000).

Cdc20 overexpression prevents the SAC from inhibiting the APC/C, leading to mitotic slippage (Bonaiuti et al., 2018). Persistent SAC-activation due to an incorrect spindle assembly may induce mitotic arrest (Kapanidou et al., 2017). Misregulation of the APC/C may allow cells to pass this cell cycle checkpoint and to proliferate uncontrolled (Riffell et al., 2009), leading to chromosomal aberrations (Zhu et al., 2014) and probably resistance to chemotherapeutic agents that interfere with the microtubules of the mitotic spindle (Liu et al., 2019). Thus, Cdc20 acts as an oncogene (Schrock et al., 2020). A correlation between higher Cdc20 expression and poorer prognosis has been demonstrated in various malignant tumors such as breast or non-small cell lung cancer (NSCLC) (Kato et al., 2012; Karra et al., 2014). Increased levels of Cdc20 lead to a dysregulated cell cycle by overwhelming the inhibitory capacity of the SAC (Izawa and Pines, 2015), thus APC/C-activation despite an active SAC may cause premature mitotic exit, resulting in dysregulated proliferation and tumorigenesis (Bonaiuti et al., 2018). A dysfunction of the APC/C itself can lead to accumulation of Cdc20 due to its inefficient degradation. Cdc20 overexpression is accompanied by the overexpression of various other genes associated with APC/C impairment in diverse cancers (Zhang et al., 2019), including overexpression of other APC/C substrates, indicating that impairment of the APC/C and not specifically Cdc20 overexpression is important for tumorigenesis.

## The Role of Cdh1 in Cell Differentiation

The APC/C^Cdh1^ is one of the most important cell cycle regulators in G1/G0-phase, where the decision between entry into a new division cycle or terminal differentiation after cell cycle exit is made. Thus, it does not only play a role in maintaining genomic stability but also in regulating cell differentiation. Its role in differentiation processes has been described in several cell types, such as neurons, myocytes and hepatocytes (Wirth et al., 2004; Li et al., 2007; Delgado-Esteban et al., 2013). In hematopoietic stem cells (HSC), a tightly controlled cell cycle regulation is crucial for the balance between differentiation and self-renewal and impairment can lead to leukemogenesis and clonal expansion of leukemic blasts. Cdh1-expression is high in human CD34-positive HSC and declines after initiation of differentiation and Cdh1-kd inhibits myeloid differentiation, contributes to B-cell development and the preservation of immature HSC without affecting proliferation or viability (Ewerth et al., 2019). The significantly decreased Cdh1-expression in blasts of acute myeloid leukemia (AML) as compared to HSC may be a possible cause of their differentiation block (Ewerth et al., 2016). In contrast, acute promyelocytic leukemia (APL) blasts are resistant to differentiation block mediated by low Cdh1-expression. Here, Cdh1-depletion leads to a marked decrease in cell viability upon all-trans retinoic acid (ATRA)-induced differentiation (Ewerth et al., 2016). Thus, low levels of Cdh1 may enhance the therapeutic effect of ATRA. In APL, the differentiation block is primarily caused by differentiation genes downregulated *via* the PML-RARα fusion gene and modulation of ubiquitination *via* APC/C^Cdh1^ appears ineffective. By inducing myeloid differentiation, ATRA can lead to long-term remissions in APL, depending on the risk constellation in combination with other agents (Sanz et al., 2009). In contrast, differentiation by PMA is controlled by both gene regulation and proteasomal degradation and PMA-stimulation of non-APL myeloid blasts leads to an increased Cdh1-expression (Li et al., 2014). After Cdh1-kd, differentiation in these cells may be delayed by Id2 stabilization but proliferation is not disrupted. In contrast, differentiation in APL is not perturbed after Cdh1-kd. Thus, ATRA here possibly leads to differentiated but genomically unstable cells due to low Cdh1 expression, with a consequently increased apoptosis rate (Ewerth et al., 2016). In solid tumor cells, Cdh1-kd led to a higher susceptibility to replication stress by DNA-damage-inducing chemotherapy (Wheeler et al., 2008) which may also explain the high efficacy of anthracyclines in combination with ATRA in APL with low Cdh1-expression. Cdh1 levels are probably controlled post-transcriptionally in AML by SCF-mediated proteolysis (Fukushima et al., 2013). The SCF-subunit Skp2 plays a role in tumorigenesis and -growth, Skp2-overexpression has also been demonstrated in AML (Min et al., 2004). In Skp2-kd cells, elevated Cdh1 levels were detected (Ewerth et al., 2016). Conversely, Skp2 is also a Cdh1 substrate: As described above, Cdh1-mediated Skp2-degradation in early G1-phase leads to stabilization of the Cdk-inhibitors p21 and p27; after Cdh1 inactivation in late G1-phase, Skp2 stabilization then leads to degradation of p21, p27, and Cdh1 and to S-phase entry (Bashir et al., 2004; Wäsch and Engelbert, 2005; Yuan et al., 2014).

## Therapeutic Approaches Targeting the APC/C

**FIGURE 2:**
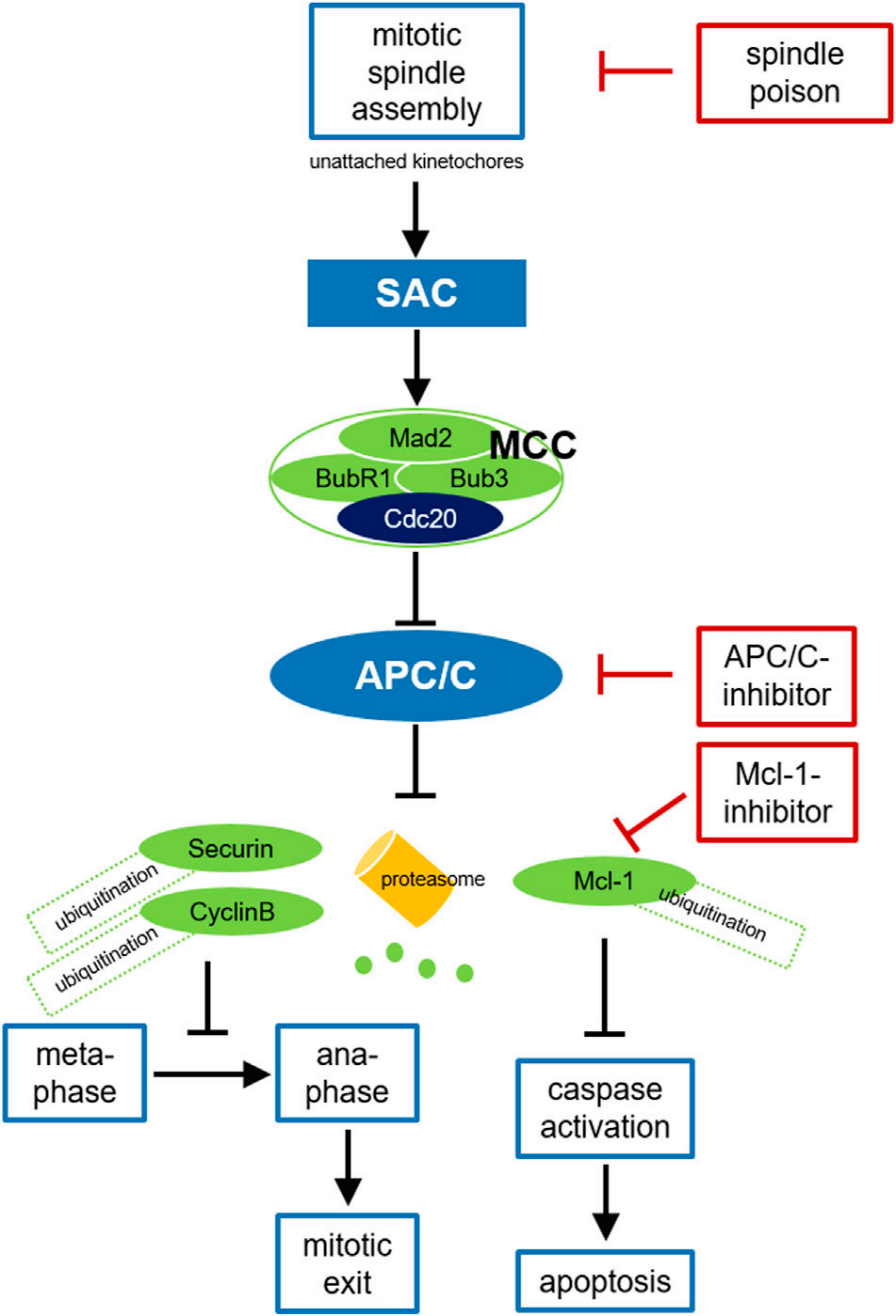
Cancer treatment targeting the APC/C. Due to disturbed microtubule kinetics after spindle poison treatment with resulting defective chromosome attachment and free kinetochores, the SAC cannot be satisfied and the MCC is formed. After binding to Mad2, BubR1, and Bub3 in the MCC Cdc20 can no longer activate the APC/C. APC/C inhibition leads to stabilization of securin and cyclin B, preventing chromosome segregation and mitotic exit. Prolonged mitotic arrest triggers Mcl-1-degradation, thus caspase activation and apoptosis in mitosis. SAC-deficient malignant cells may survive treatment with spindle poisons due to residual APC/C activity and slow cyclin B-degradation. Inhibition of APC/C or the proteasome can prevent this mitotic slippage, consolidate the mitotic block and enhance the antitumor effect. Mcl-1 inhibition may additionally promote apoptosis. SAC, spindle assembly checkpoint; MCC, mitotic checkpoint complex; APC/C, anaphase promoting complex.

Treatment with spindle poisons like taxanes and vinca alkaloids which inhibit the assembly of the mitotic spindle belong to the most common chemotherapeutic approaches in various malignant solid tumors (Dominguez-Brauer et al., 2015). Paclitaxel stabilizes the microtubule polymer and protects it from disassembly. It could be shown that high doses lead to a sustained mitotic arrest after SAC-activation (Weaver, 2014): due to the suppression of microtubule dynamics, chromosomes are unable to achieve a metaphase spindle configuration which prevents the SAC from being satisfied (Stukenberg and Burke, 2015), leading to continuous APC/C-inhibition with persistent high levels of cyclin B and mitotic arrest to provide additional time to repair the spindle damage (Figure 2). If this attempt fails, SAC-induced mitotic arrest triggers either apoptosis in mitosis *via* the mitochondrial pathway (Topham and Taylor, 2013), leads to reversion to the G0-phase and dormancy without cell division or results in mitotic slippage with mitotic exit prior to a successful cell division and thus to G1-entry of tetraploid cells. These cells may either die right after G1-entry or re-enter the cell cycle leading to proliferation of cells with an aberrant genotype (Fujiwara et al., 2005; Gascoigne and Taylor, 2008). However, other studies have suggested that therapeutically relevant concentrations of paclitaxel may not lead to mitotic arrest but to multipolar spindles and thus to chromosome missegregation and increased cell death in the interphase that follows the perturbed mitosis (Zasadil et al., 2014; Zeng et al., 2019). It was shown in several tumor cell lines that spindle apparatus damage can still result in mitotic exit without apoptosis in mitosis (Shi et al., 2008). For example in breast cancer cells, *in vivo* achievable paclitaxel concentrations led to multipolar spindles, incorrect chromosome distribution and postmitotic cell death (Zasadil et al., 2014; Alves et al., 2018).

The mechanism mediating whether a cell survives prolonged mitosis after spindle damage or enters apoptosis is not fully understood. A model with two independent competing signaling pathways is discussed: During mitotic arrest, pro-apoptotic signals continuously increase while cyclin B levels decrease. If the cyclin B level falls below the threshold that allows mitotic escape first, mitotic slippage occurs; if the threshold for apoptosis initiation is crossed first, mitotic cell death is initiated (Gascoigne and Taylor, 2008; Topham and Taylor, 2013). Thus, two mechanisms may contribute to the survival of tumor cells despite perturbed mitotic spindle assembly and despite subsequent mitotic arrest and thus to reduced efficacy of spindle poisons (Gascoigne and Taylor, 2008): a predominance of mitotic slippage or a dysregulation of the apoptosis signaling pathway.

Nevertheless, tumor cells can escape a SAC-mediated mitotic arrest by mitotic slippage (Brito and Rieder, 2006; Gascoigne and Taylor, 2008). Even if the SAC cannot be passed after spindle damage, residual background APC/C-activity may result in slow but continuous cyclin B-degradation *via* the ubiquitin-proteasome system (Brito and Rieder, 2006; Schnerch et al., 2012b). If the cyclin B level falls below a critical limit, mitotic arrest can no longer be maintained although the SAC has not been satisfied. This prevents initiation of apoptosis after prolonged mitosis and may reduce the efficacy of spindle poisons, comparable to a premature mitotic exit due to a weakened or abolished SAC (Gascoigne and Taylor, 2008). Consequently, preventing exit from mitosis by APC/C-inhibition when the SAC is not satisfied should enhance the antitumor effect (Figure 2). Some data indicate that the APC/C and its coactivator Cdc20 could be targets for oncological therapies. Cdc20-kd leads to mitotic arrest and apoptosis in various tumor cell lines (Huang et al., 2009). In 2014, the efficacy of direct APC/C-inhibitors was described for the first time: By binding Cdc20, apcin competitively prevents substrate recognition. The efficacy of apcin in blocking mitotic exit is synergistically amplified by proTAME (tosyl-L-arginine methyl ester), a prodrug that is converted to TAME by an intracellular esterase and blocks Cdc20 binding to the APC/C at a different site (Sackton et al., 2014). The combination of both molecules (pT/A) thus leads to a significant stabilization of APC/C-substrates, prevents mitotic exit in tumor cells and promotes apoptosis (Sackton et al., 2014). APC/C-inhibitors have not yet been tested in clinical trials, but preclinical data suggest a promising therapeutic approach: For example, it has been shown that proTAME leads to enhanced mitotic arrest and apoptosis in paclitaxel-treated cell lines of various solid tumors like ovarian cancer (Zeng et al., 2010; Giovinazzi et al., 2013; Sinnott et al., 2014; Raab et al., 2019). pT/A can reduce the viability of glioblastoma cells (De et al., 2019) and shows also activity in hematologic neoplasms like multiple myeloma and different types of non-Hodgkin lymphoma (Lub et al., 2016; Maes et al., 2019). Since slow cyclin B-degradation is mediated by the proteasome, preventing mitotic slippage by inhibiting proteasomal degradation seems reasonable. Indeed, a stable mitotic block was demonstrated after treatment with a proteasome inhibitor (Brito and Rieder, 2006; Schnerch et al., 2012b). Programmed cell death *via* the intrinsic signaling pathway is triggered by cellular stress following defective cell division or by insufficient repair mechanisms and is essential for the destruction of degenerate or potentially harmful cells (Green and Llambi, 2015). In response to DNA-damage, pro-apoptotic members of the Bcl-2 family such as BAD and Bim are activated and can thus neutralize anti-apoptotic members such as Bcl-2 itself, Bcl-xL, and Mcl-1, leading to mitochondrial outer membrane permeabilization *via* the pore-forming proteins BAX and BAK, to the release of various effector proteins such as cytochrome C, to caspase activation and cleavage of cellular proteins (Kalkavan and Green, 2018). Indeed, it has been shown that cell death during a mitotic arrest is initiated *via* this mitochondrial pathway (Gascoigne and Taylor, 2008). An imbalance between members of the Bcl-2 family may result in inefficient apoptosis (Delbridge et al., 2016). Because of the complexity of these signaling pathways, it is challenging to identify the key regulators that trigger apoptosis during prolonged mitosis. During prolonged mitotic arrest, nearly all Bcl-2 family proteins and caspases are posttranscriptionally modified (Topham and Taylor, 2013). The protein Mcl-1, which is overexpressed in many tumors, seems to play a central role here (Akgul, 2009; Beroukhim et al., 2010; Harley et al., 2010). Mcl-1 levels are regulated in a cell cycle-dependent manner, being highest in G2-phase. During mitosis, Mcl-1 is degraded after Cdk1/cyclin B-mediated phosphorylation *via* APC/C^Cdc20^ (Figure 2). Due to the slow degradation of this anti-apoptotic protein, cells arrested in mitosis after therapy with spindle poisons can initiate apoptosis (Harley et al., 2010). Conversely, stabilization of Mcl-1 during mitotic arrest, for example by mutation of phosphorylation sites, prevents initiation of apoptosis after antimitotic therapy. In various tumors it was shown, that small molecules inhibiting anti-apoptotic proteins such as Mcl-1 reactivate the apoptosis signaling pathway (Wäsch, 2011; Leverson et al., 2015; Raab et al., 2019).

Besides conventional chemotherapy, those small molecules targeting signaling pathways that can be deregulated in tumor cells currently play an increasing role, as they may be more effective and cause fewer side effects. Inhibitors directed against cell cycle regulators such as Plk1 are in preclinical development and are tested in clinical trials (Gutteridge et al., 2016; Zeidan et al., 2020). Mitotic block after antimitotic therapy with classical spindle poisons or with such small molecules should be consolidated by preventing mitotic slippage through slow cyclin B-degradation *via* APC/C- or proteasome inhibition, thus enhancing the antitumor effect. Lower levels of the APC/C-coactivator Cdc20 reduced cyclin B-degradation even after mitotic arrest by spindle poison treatment (Schnerch et al., 2017). The effect of antimitotic therapy could be enhanced by additional proteasome inhibition in AML (Schnerch et al., 2017). It was suggested that the proteasome inhibitor bortezomib leads to G2/M-arrest in myeloid blasts and thus acts synergistically with antimitotic agents (Colado et al., 2008; Bucur et al., 2013). Nevertheless, it was shown that bortezomib acts primarily in mitosis, prevents slow cyclin B degradation and cyclin B overexpression enhances mitotic arrest after volasertib (Schnerch et al., 2017). Delayed mitotic progression after cyclin B overexpression was described (Gascoigne and Taylor, 2008) and a similar effect in volasertib-treated cells by adding bortezomib (Schnerch et al., 2017). Paclitaxel and bortezomib have also been used sequentially in solid tumors at low concentrations achievable *in vivo*(Weaver, 2014; Weyburne et al., 2017). *In vitro*, a similar effect of proteasome inhibitors on solid tumor cells as on myeloma cells was observed (Garnett et al., 2012). The sequential combination of antimitotic agents with proteasome inhibition enhances cell death in different solid tumor cells, but during interphase and not through the presumed effect of a consolidated mitotic arrest with subsequent apoptosis in mitosis (Greil et al., 2021). In clinical trials, neither bortezomib alone nor a combination with doxorubicin has been shown to have a therapeutic effect in lung and breast cancer (Dou and Zonder, 2014), perhaps because the bortezomib concentration achievable *in vivo* inhibits only one of the three subunits of the proteasome (Weyburne et al., 2017).

Consistent with the presumed synergistic effect on mitotic exit, APC/C-inhibition alone or combined with a taxane can lead to strong mitotic cell death in certain tumor cells, but in others with high expression of the anti-apoptotic regulator Mcl-1, cell death is induced in interphase (Zeng et al., 2010; Raab et al., 2020; Greil et al., 2021). Here, combined APC/C- and Mcl-1-inhibition is highly lethal but also in interphase (Greil et al., 2021). Despite an initially increased apoptosis rate and reduced cell number shortly after exposure, the substances do not always lead to a durable response in a cell type-specific manner (Greil et al., 2021). This difference between short-term effect and long-term survival seems to be explained by mitotic slippage (Zeng et al., 2019): These cells escape mitotic block and thus evade cell death shortly after spindle toxin therapy, but their long-term survival is compromised by increasing genomic instability after disrupted cell division.

## Conclusion

The APC/C either has an oncogenic function after binding to its co-activator Cdc20, or works as a tumor suppressor when bound to Cdh1. Thus, dysfunction of both the APC/C^Cdh1^ and APC/C^Cdc20^ and loss of Cdh1 itself lead to dysregulation of the cell cycle, aneuploidy and increased genomic instability and contribute to tumorigenesis and probably drug resistance.

Targeting the APC/C has shown potent anti-tumor capacity and the combination of spindle poisons with a proteasome inhibitor or direct inhibitors of the APC/C and Mcl-1 seems a promising approach to improve treatment response in different solid tumors, even though they act entity-dependent at different cell cycle phases. Taken together, the current state of knowledge provides a compelling rationale to further pursue on the role of the APC/C in tumor development and treatment.

## References

[B1] Akgul C. (2009). Mcl-1 Is a Potential Therapeutic Target in Multiple Types of Cancer. Cell. Mol. Life Sci. 66, 1326–1336. 10.1007/s00018-008-8637-6 19099185 PMC11131550

[B2] Alves R. C. Fernandes R. P. Eloy J. O. Salgado H. R. N. Chorilli M. (2018). Characteristics, Properties and Analytical Methods of Paclitaxel: A Review. Crit. Rev. Anal. Chem. 48, 110–118. 10.1080/10408347.2017.1416283 29239659

[B3] Ayuda-Duran P. Devesa F. Gomes F. Sequeira-Mendes J. Avila-Zarza C. Gomez M. (2014). The CDK Regulators Cdh1 and Sic1 Promote Efficient Usage of DNA Replication Origins to Prevent Chromosomal Instability at a Chromosome Arm. Nucleic Acids Res. 42, 7057–7068. 10.1093/nar/gku313 24753426 PMC4066753

[B4] Bashir T. Dorrello N. V. Amador V. Guardavaccaro D. Pagano M. (2004). Control of the SCFSkp2-Cks1 Ubiquitin Ligase by the APC/CCdh1 Ubiquitin Ligase. Nature 428, 190–193. 10.1038/nature02330 15014502

[B5] Bassermann F. Frescas D. Guardavaccaro D. Busino L. Peschiaroli A. Pagano M. (2008). The Cdc14B-Cdh1-Plk1 Axis Controls the G2 DNA-Damage-Response Checkpoint. Cell 134, 256–267. 10.1016/j.cell.2008.05.043 18662541 PMC2591934

[B6] Beroukhim R. Mermel C. H. Porter D. Wei G. Raychaudhuri S. Donovan J. (2010). The Landscape of Somatic Copy-Number Alteration across Human Cancers. Nature 463, 899–905. 10.1038/nature08822 20164920 PMC2826709

[B7] Bonaiuti P. Chiroli E. Gross F. Corno A. Vernieri C. Štefl M. (2018). Cells Escape an Operational Mitotic Checkpoint through a Stochastic Process. Curr. Biol. 28, 28–37. e7. 10.1016/j.cub.2017.11.031 29249657

[B8] Brito D. A. Rieder C. L. (2006). Mitotic Checkpoint Slippage in Humans Occurs via Cyclin B Destruction in the Presence of an Active Checkpoint. Curr. Biol. 16, 1194–1200. 10.1016/j.cub.2006.04.043 16782009 PMC2749311

[B9] Bucur O. Stancu A. L. Goganau I. Petrescu S. M. Pennarun B. Bertomeu T. (2013). Combination of Bortezomib and Mitotic Inhibitors Down-Modulate Bcr-Abl and Efficiently Eliminates Tyrosine-Kinase Inhibitor Sensitive and Resistant Bcr-Abl-Positive Leukemic Cells. PLOS ONE 8, e77390. 10.1371/journal.pone.0077390 24155950 PMC3796452

[B10] Choudhury R. Bonacci T. Arceci A. Lahiri D. Mills C. A. Kernan J. L. (2016). APC/C and SCF Cyclin F Constitute a Reciprocal Feedback Circuit Controlling S-phase Entry. Cell Rep. 16, 3359–3372. 10.1016/j.celrep.2016.08.058 27653696 PMC5111906

[B11] Colado E. Alvarez-Fernandez S. Maiso P. Martin-Sanchez J. Vidriales M. B. Garayoa M. (2008). The Effect of the Proteasome Inhibitor Bortezomib on Acute Myeloid Leukemia Cells and Drug Resistance Associated with the CD34+ Immature Phenotype. Haematologica 93, 57–66. 10.3324/haematol.11666 18166786

[B12] de Boer H. R. Guerrero Llobet S. van Vugt M. A. T. M. (2016). Controlling the Response to DNA Damage by the APC/C-Cdh1. Cell. Mol. Life Sci. 73, 949–960. 10.1007/s00018-015-2096-7 26650195 PMC4744251

[B13] De K. Grubb T. M. Zalenski A. A. Pfaff K. E. Pal D. Majumder S. (2019). Hyperphosphorylation of CDH1 in Glioblastoma Cancer Stem Cells Attenuates APC/CCDH1 Activity and Pharmacologic Inhibition of APC/CCDH1/CDC20 Compromises Viability. Mol. Cancer Res. 17, 1519–1530. 10.1158/1541-7786.MCR-18-1361 31036696 PMC7271747

[B14] Delbridge A. R. D. Grabow S. Strasser A. Vaux D. L. (2016). Thirty Years of BCL-2: Translating Cell Death Discoveries into Novel Cancer Therapies. Nat. Rev. Cancer 16, 99–109. 10.1038/nrc.2015.17 26822577

[B15] Delgado-Esteban M. García-Higuera I. Maestre C. Moreno S. Almeida A. (2013). APC/C-Cdh1 Coordinates Neurogenesis and Cortical Size during Development. Nat. Commun. 4, 2879. 10.1038/ncomms3879 24301314

[B16] Diffley J. F. X. (2004). Regulation of Early Events in Chromosome Replication. Curr. Biol. 14, R778–R786. 10.1016/j.cub.2004.09.019 15380092

[B17] Dominguez-Brauer C. Thu K. L. Mason J. M. Blaser H. Bray M. R. Mak T. W. (2015). Targeting Mitosis in Cancer: Emerging Strategies. Mol. Cell 60, 524–536. 10.1016/j.molcel.2015.11.006 26590712

[B18] Dou Q. Zonder J. (2014). Overview of Proteasome Inhibitor-Based Anti-cancer Therapies: Perspective on Bortezomib and Second Generation Proteasome Inhibitors versus Future Generation Inhibitors of Ubiquitin-Proteasome System. Ccdt 14, 517–536. 10.2174/1568009614666140804154511 PMC4279864 25092212

[B19] Drosopoulos K. Tang C. Chao W. C. H. Linardopoulos S. (2014). APC/C Is an Essential Regulator of Centrosome Clustering. Nat. Commun. 5, 3686. 10.1038/ncomms4686 24751481

[B20] Eguren M. Álvarez-Fernández M. García F. López-Contreras A. J. Fujimitsu K. Yaguchi H. (2014). A Synthetic Lethal Interaction between APC/C and Topoisomerase Poisons Uncovered by Proteomic Screens. Cell Rep. 6, 670–683. 10.1016/j.celrep.2014.01.017 24508461

[B21] Engelbert D. Schnerch D. Baumgarten A. Wäsch R. (2008). The Ubiquitin Ligase APCCdh1 Is Required to Maintain Genome Integrity in Primary Human Cells. Oncogene 27, 907–917. 10.1038/sj.onc.1210703 17700535

[B22] Evans T. Rosenthal E. T. Youngblom J. Distel D. Hunt T. (1983). Cyclin: A Protein Specified by Maternal MRNA in Sea Urchin Eggs that Is Destroyed at Each Cleavage Division. Cell 33, 389–396. 10.1016/0092-8674(83)90420-8 6134587

[B23] Ewerth D. Kreutmair S. Schmidts A. Ihorst G. Follo M. Wider D. (2019). APC/CCdh1 Regulates the Balance between Maintenance and Differentiation of Hematopoietic Stem and Progenitor Cells. Cell. Mol. Life Sci. 76, 369–380. 10.1007/s00018-018-2952-3 30357422 PMC11105657

[B24] Ewerth D. Schmidts A. Hein M. Schnerch D. Kvainickas A. Greil C. (2016). Suppression of APC/CCdh1 Has Subtype Specific Biological Effects in Acute Myeloid Leukemia. Oncotarget 7, 48220–48230. 10.18632/oncotarget.10196 27374082 PMC5217013

[B25] Foley E. A. Kapoor T. M. (2013). Microtubule Attachment and Spindle Assembly Checkpoint Signalling at the Kinetochore. Nat. Rev. Mol. Cell Biol. 14, 25–37. 10.1038/nrm3494 23258294 PMC3762224

[B26] Fujiwara T. Bandi M. Nitta M. Ivanova E. V. Bronson R. T. Pellman D. (2005). Cytokinesis Failure Generating Tetraploids Promotes Tumorigenesis in P53-Null Cells. Nature 437, 1043–1047. 10.1038/nature04217 16222300

[B27] Fukushima H. Ogura K. Wan L. Lu Y. Li V. Gao D. (2013). SCF-mediated Cdh1 Degradation Defines a Negative Feedback System that Coordinates Cell-Cycle Progression. Cell Rep. 4, 803–816. 10.1016/j.celrep.2013.07.031 23972993 PMC3839583

[B28] Ganem N. J. Godinho S. A. Pellman D. (2009). A Mechanism Linking Extra Centrosomes to Chromosomal Instability. Nature 460, 278–282. 10.1038/nature08136 19506557 PMC2743290

[B29] García-Higuera I. Manchado E. Dubus P. Cañamero M. Méndez J. Moreno S. (2008). Genomic Stability and Tumour Suppression by the APC/C Cofactor Cdh1. Nat. Cell Biol. 10, 802–811. 10.1038/ncb1742 18552834

[B30] Garnett M. J. Edelman E. J. Heidorn S. J. Greenman C. D. Dastur A. Lau K. W. (2012). Systematic Identification of Genomic Markers of Drug Sensitivity in Cancer Cells. Nature 483, 570–575. 10.1038/nature11005 22460902 PMC3349233

[B31] Gascoigne K. E. Taylor S. S. (2008). Cancer Cells Display Profound Intra- and Interline Variation Following Prolonged Exposure to Antimitotic Drugs. Cancer Cell 14, 111–122. 10.1016/j.ccr.2008.07.002 18656424

[B32] Giovinazzi S. Bellapu D. Morozov V. M. Ishov A. M. (2013). Targeting Mitotic Exit with Hyperthermia or APC/C Inhibition to Increase Paclitaxel Efficacy. Cell Cycle 12, 2598–2607. 10.4161/cc.25591 23907120 PMC3865049

[B33] Green D. R. Llambi F. (2015). Cell Death Signaling. Cold Spring Harb. Perspect. Biol. 7, a006080. 10.1101/cshperspect.a006080 26626938 PMC4665079

[B34] Greil C. Felthaus J. Follo M. Ihorst G. Ewerth D. Schüler J. (2021). Targeting Mitotic Exit in Solid Tumors. Am. J. Cancer Res. 11, 3698–3710. 34354869 PMC8332852

[B35] Greil C. Krohs J. Schnerch D. Follo M. Felthaus J. Engelhardt M. (2016). The Role of APC/CCdh1 in Replication Stress and Origin of Genomic Instability. Oncogene 35, 3062–3070. 10.1038/onc.2015.367 26455319

[B36] Gutteridge R. E. A. Ndiaye M. A. Liu X. Ahmad N. (2016). Plk1 Inhibitors in Cancer Therapy: From Laboratory to Clinics. Mol. Cancer Ther. 15, 1427–1435. 10.1158/1535-7163.MCT-15-0897 27330107 PMC4936921

[B37] Han J. S. Holland A. J. Fachinetti D. Kulukian A. Cetin B. Cleveland D. W. (2013). Catalytic Assembly of the Mitotic Checkpoint Inhibitor BubR1-Cdc20 by a Mad2-Induced Functional Switch in Cdc20. Mol. Cell 51, 92–104. 10.1016/j.molcel.2013.05.019 23791783 PMC3713096

[B38] Harley M. E. Allan L. A. Sanderson H. S. Clarke P. R. (2010). Phosphorylation of Mcl-1 by CDK1-Cyclin B1 Initiates its Cdc20-dependent Destruction during Mitotic Arrest. EMBO J. 29, 2407–2420. 10.1038/emboj.2010.112 20526282 PMC2910263

[B39] Harper J. W. Burton J. L. Solomon M. J. (2002). The Anaphase-Promoting Complex: It's Not Just for Mitosis Any More. Genes Dev. 16, 2179–2206. 10.1101/gad.1013102 12208841

[B40] Hershko A. Ciechanover A. (1998). The Ubiquitin System. Annu. Rev. Biochem. 67, 425–479. 10.1146/annurev.biochem.67.1.425 9759494

[B41] Huang H.-C. Shi J. Orth J. D. Mitchison T. J. (2009). Evidence that Mitotic Exit Is a Better Cancer Therapeutic Target Than Spindle Assembly. Cancer Cell 16, 347–358. 10.1016/j.ccr.2009.08.020 19800579 PMC2758291

[B42] Ishizawa J. Kuninaka S. Sugihara E. Naoe H. Kobayashi Y. Chiyoda T. (2011). The Cell Cycle Regulator Cdh1 Controls the Pool Sizes of Hematopoietic Stem Cells and Mature Lineage Progenitors by Protecting from Genotoxic Stress. Cancer Sci. 102, 967–974. 10.1111/j.1349-7006.2011.01884.x 21255192

[B43] Izawa D. Pines J. (2015). The Mitotic Checkpoint Complex Binds a Second CDC20 to Inhibit Active APC/C. Nature 517, 631–634. 10.1038/nature13911 25383541 PMC4312099

[B44] Jallepalli P. V. Lengauer C. (2001). Chromosome Segregation and Cancer: Cutting through the Mystery. Nat. Rev. Cancer 1, 109–117. 10.1038/35101065 11905802

[B45] Kalkavan H. Green D. R. (2018). MOMP, Cell Suicide as a BCL-2 Family Business. Cell Death Differ. 25, 46–55. 10.1038/cdd.2017.179 29053143 PMC5729535

[B46] Kapanidou M. Curtis N. L. Bolanos-Garcia V. M. (2017). Cdc20: At the Crossroads between Chromosome Segregation and Mitotic Exit. Trends Biochem. Sci. 42, 193–205. 10.1016/j.tibs.2016.12.001 28202332

[B47] Karra H. Repo H. Ahonen I. Löyttyniemi E. Pitkänen R. Lintunen M. (2014). Cdc20 and Securin Overexpression Predict Short-Term Breast Cancer Survival. Br. J. Cancer 110, 2905–2913. 10.1038/bjc.2014.252 24853182 PMC4056061

[B48] Kato T. Daigo Y. Aragaki M. Ishikawa K. Sato M. Kaji M. (2012). Overexpression of CDC20 Predicts Poor Prognosis in Primary Non-small Cell Lung Cancer Patients. J. Surg. Oncol. 106, 423–430. 10.1002/jso.23109 22488197

[B49] King R. W. Deshaies R. J. Peters J.-M. Kirschner M. W. (1996). How Proteolysis Drives the Cell Cycle. Science 274, 1652–1659. 10.1126/science.274.5293.1652 8939846

[B51] Lawrence K. S. Chau T. Engebrecht J. (2015). DNA Damage Response and Spindle Assembly Checkpoint Function throughout the Cell Cycle to Ensure Genomic Integrity. PLoS Genet. 11, e1005150. 10.1371/journal.pgen.1005150 25898113 PMC4405263

[B52] Leverson J. D. Zhang H. Chen J. Tahir S. K. Phillips D. C. Xue J. (2015). Potent and Selective Small-Molecule MCL-1 Inhibitors Demonstrate On-Target Cancer Cell Killing Activity as Single Agents and in Combination with ABT-263 (Navitoclax). Cell Death Dis. 6, e1590. 10.1038/cddis.2014.561 25590800 PMC4669759

[B53] Li C. Peart N. Xuan Z. Lewis D. E. Xia Y. Jin J. (2014). PMA Induces SnoN Proteolysis and CD61 Expression through an Autocrine Mechanism. Cell. Signal. 26, 1369–1378. 10.1016/j.cellsig.2014.03.006 24637302 PMC4074601

[B54] Li W. Wu G. Wan Y. (2007). The Dual Effects of Cdh1/APC in Myogenesis. FASEB J. 21, 3606–3617. 10.1096/fj.07-8159com 17601983

[B55] Liu X. Chen Y. Li Y. Petersen R. B. Huang K. (2019). Targeting Mitosis Exit: A Brake for Cancer Cell Proliferation. Biochimica Biophysica Acta (BBA) - Rev. Cancer 1871, 179–191. 10.1016/j.bbcan.2018.12.007 30611728

[B56] Lub S. Maes A. Maes K. De Veirman K. De Bruyne E. Menu E. (2016). Inhibiting the Anaphase Promoting Complex/Cyclosome Induces a Metaphase Arrest and Cell Death in Multiple Myeloma Cells. Oncotarget 7, 4062–4076. 10.18632/oncotarget.6768 26716651 PMC4826190

[B57] Maes A. Maes K. De Raeve H. De Smedt E. Vlummens P. Szablewski V. (2019). The Anaphase-Promoting Complex/Cyclosome: A New Promising Target in Diffuse Large B-Cell Lymphoma and Mantle Cell Lymphoma. Br. J. Cancer 120, 1137–1146. 10.1038/s41416-019-0471-0 31089208 PMC6738099

[B58] Miller M. E. Cross F. R. (2001). Cyclin Specificity: How Many Wheels Do You Need on a Unicycle? J. Cell Sci. 114, 1811–1820. 10.1242/jcs.114.10.1811 11329367

[B59] Min Y. H. Cheong J.-W. Lee M. H. Kim J. Y. Lee S. T. Hahn J. S. (2004). Elevated S-phase Kinase-Associated Protein 2 Protein Expression in Acute Myelogenous Leukemia. Clin. Cancer Res. 10, 5123–5130. 10.1158/1078-0432.CCR-04-0136 15297415

[B60] Morgan D. O. (1999). Regulation of the APC and the Exit from Mitosis. Nat. Cell Biol. 1, E47–E53. E53. 10.1038/10039 10559897

[B61] Musacchio A. Salmon E. D. (2007). The Spindle-Assembly Checkpoint in Space and Time. Nat. Rev. Mol. Cell Biol. 8, 379–393. 10.1038/nrm2163 17426725

[B62] Musacchio A. (2015). The Molecular Biology of Spindle Assembly Checkpoint Signaling Dynamics. Curr. Biol. 25, R1002–R1018. 10.1016/j.cub.2015.08.051 26485365

[B63] Neelsen K. J. Zanini I. M. Y. Herrador R. Lopes M. (2013). Oncogenes Induce Genotoxic Stress by Mitotic Processing of Unusual Replication Intermediates. J. Cell Biol. 200, 699–708. 10.1083/jcb.201212058 23479741 PMC3601361

[B64] Negrini S. Gorgoulis V. G. Halazonetis T. D. (2010). Genomic Instability - an Evolving Hallmark of Cancer. Nat. Rev. Mol. Cell Biol. 11, 220–228. 10.1038/nrm2858 20177397

[B65] Peters J.-M. (2006). The Anaphase Promoting Complex/Cyclosome: A Machine Designed to Destroy. Nat. Rev. Mol. Cell Biol. 7, 644–656. 10.1038/nrm1988 16896351

[B66] Pines J. (2011). Cubism and the Cell Cycle: The Many Faces of the APC/C. Nat. Rev. Mol. Cell Biol. 12, 427–438. 10.1038/nrm3132 21633387

[B67] Qiao X. Zhang L. Gamper A. M. Fujita T. Wan Y. (2010). APC/C-Cdh1. Cell Cycle 9, 3904–3912. 10.4161/cc.9.19.13585 20935501 PMC3047751

[B68] Raab M. Kobayashi N. F. Becker S. Kurunci‐Csacsko E. Krämer A. Strebhardt K. (2020). Boosting the Apoptotic Response of High‐grade Serous Ovarian Cancers with CCNE1 Amplification to Paclitaxel *In Vitro* by Targeting APC/C and the Pro‐survival Protein MCL‐1. Int. J. Cancer 146, 1086–1098. 10.1002/ijc.32559 31286496

[B69] Raab M. Sanhaji M. Zhou S. Rödel F. El-Balat A. Becker S. (2019). Blocking Mitotic Exit of Ovarian Cancer Cells by Pharmaceutical Inhibition of the Anaphase-Promoting Complex Reduces Chromosomal Instability. Neoplasia 21, 363–375. 10.1016/j.neo.2019.01.007 30851646 PMC6407080

[B70] Riffell J. L. Zimmerman C. Khong A. McHardy L. M. Roberge M. (2009). Effects of Chemical Manipulation of Mitotic Arrest and Slippage on Cancer Cell Survival and Proliferation. Cell Cycle 8, 3025–3038. 10.4161/cc.8.18.9623 19713760

[B71] Rouse J. Jackson S. P. (2002). Interfaces between the Detection, Signaling, and Repair of DNA Damage. Science 297, 547–551. 10.1126/science.1074740 12142523

[B72] Sackton K. L. Dimova N. Zeng X. Tian W. Zhang M. Sackton T. B. (2014). Synergistic Blockade of Mitotic Exit by Two Chemical Inhibitors of the APC/C. Nature 514, 646–649. 10.1038/nature13660 25156254 PMC4214887

[B73] Sanz M. A. Grimwade D. Tallman M. S. Lowenberg B. Fenaux P. Estey E. H. (2009). Management of Acute Promyelocytic Leukemia: Recommendations from an Expert Panel on Behalf of the European LeukemiaNet. Blood 113, 1875–1891. 10.1182/blood-2008-04-150250 18812465

[B74] Schnerch D. Yalcintepe J. Schmidts A. Becker H. Follo M. Engelhardt M. (2012). Cell Cycle Control in Acute Myeloid Leukemia. Am. J. Cancer Res. 2, 508–528. 22957304 PMC3433102

[B75] Schnerch D. Follo M. Krohs J. Felthaus J. Engelhardt M. Wäsch R. (2012). Monitoring APC/C Activity in the Presence of Chromosomal Misalignment in Unperturbed Cell Populations. Cell Cycle 11, 310–321. 10.4161/cc.11.2.18737 22214763

[B76] Schnerch D. Schüler J. Follo M. Felthaus J. Wider D. Klingner K. (2017). Proteasome Inhibition Enhances the Efficacy of Volasertib-Induced Mitotic Arrest in AML *In Vitro* and Prolongs Survival *In Vivo* . Oncotarget 8, 21153–21166. 10.18632/oncotarget.15503 28416751 PMC5400573

[B77] Schrock M. S. Stromberg B. R. Scarberry L. Summers M. K. (2020). APC/C Ubiquitin Ligase: Functions and Mechanisms in Tumorigenesis. Seminars Cancer Biol. 67, 80–91. 10.1016/j.semcancer.2020.03.001 PMC7483777 32165320

[B78] Scully R. (2010). The Spindle-Assembly Checkpoint, Aneuploidy, and Gastrointestinal Cancer. N. Engl. J. Med. 363, 2665–2666. 10.1056/NEJMe1008017 21190461 PMC3731131

[B79] Shi J. Orth J. D. Mitchison T. (2008). Cell Type Variation in Responses to Antimitotic Drugs that Target Microtubules and Kinesin-5. Cancer Res. 68, 3269–3276. 10.1158/0008-5472.CAN-07-6699 18451153

[B80] Sinnott R. Winters L. Larson B. Mytsa D. Taus P. Cappell K. M. (2014). Mechanisms Promoting Escape from Mitotic Stress-Induced Tumor Cell Death. Cancer Res. 74, 3857–3869. 10.1158/0008-5472.CAN-13-3398 24860162 PMC4102622

[B81] Skaar J. R. Pagano M. (2008). Cdh1: a Master G0/G1 Regulator. Nat. Cell Biol. 10, 755–757. 10.1038/ncb0708-755 18591966 PMC2730193

[B82] Skinner J. J. Wood S. Shorter J. Englander S. W. Black B. E. (2008). The Mad2 Partial Unfolding Model: Regulating Mitosis through Mad2 Conformational Switching. J. Cell Biol. 183, 761–768. 10.1083/jcb.200808122 19029339 PMC2592820

[B83] Sørensen C. S. Lukas C. Kramer E. R. Peters J.-M. Bartek J. Lukas J. (2000). Nonperiodic Activity of the Human Anaphase-Promoting Complex-Cdh1 Ubiquitin Ligase Results in Continuous DNA Synthesis Uncoupled from Mitosis. Mol. Cell Biol. 20, 7613–7623. 10.1128/MCB.20.20.7613-7623.2000 11003657 PMC86321

[B84] Stukenberg P. T. Burke D. J. (2015). Connecting the Microtubule Attachment Status of Each Kinetochore to Cell Cycle Arrest through the Spindle Assembly Checkpoint. Chromosoma 124, 463–480. 10.1007/s00412-015-0515-z 25917595

[B85] Sudakin V. Chan G. K. T. Yen T. J. (2001). Checkpoint Inhibition of the APC/C in HeLa Cells Is Mediated by a Complex of BUBR1, BUB3, CDC20, and MAD2. J. Cell Biol. 154, 925–936. 10.1083/jcb.200102093 11535616 PMC2196190

[B86] Sudo T. Ota Y. Kotani S. Nakao M. Takami Y. Takeda S. (2001). Activation of Cdh1-dependent APC Is Required for G1 Cell Cycle Arrest and DNA Damage-Induced G2 Checkpoint in Vertebrate Cells. EMBO J. 20, 6499–6508. 10.1093/emboj/20.22.6499 11707420 PMC125730

[B87] Sullivan M. Morgan D. O. (2007). Finishing Mitosis, One Step at a Time. Nat. Rev. Mol. Cell Biol. 8, 894–903. 10.1038/nrm2276 17912263

[B88] Tavormina P. A. Come M.-G. Hudson J. R. Mo Y.-Y. Beck W. T. Gorbsky G. J. (2002). Rapid Exchange of Mammalian Topoisomerase IIα at Kinetochores and Chromosome Arms in Mitosis. J. Cell Biol. 158, 23–29. 10.1083/jcb.200202053 12105179 PMC2173008

[B89] Topham C. H. Taylor S. S. (2013). Mitosis and Apoptosis: How Is the Balance Set? Curr. Opin. Cell Biol. 25, 780–785. 10.1016/j.ceb.2013.07.003 23890995

[B90] Wang L. Zhang J. Wan L. Zhou X. Wang Z. Wei W. (2015). Targeting Cdc20 as a Novel Cancer Therapeutic Strategy. Pharmacol. Ther. 151, 141–151. 10.1016/j.pharmthera.2015.04.002 25850036 PMC4457591

[B91] Wäsch R. Cross F. R. (2002). APC-dependent Proteolysis of the Mitotic Cyclin Clb2 Is Essential for Mitotic Exit. Nature 418, 556–562. 10.1038/nature00856 12152084

[B92] Wäsch R. Engelbert D. (2005). Anaphase-Promoting Complex-dependent Proteolysis of Cell Cycle Regulators and Genomic Instability of Cancer Cells. Oncogene 24, 1–10. 10.1038/sj.onc.1208017 15637585

[B93] Wäsch R. Robbins J. A. Cross F. R. (2010). The Emerging Role of APC/CCdh1 in Controlling Differentiation, Genomic Stability and Tumor Suppression. Oncogene 29, 1–10. 10.1038/onc.2009.325 19826416 PMC3102600

[B94] Wäsch R. (2011). Targeting Mitotic Exit for Cancer Treatment. Expert Opin. Ther. Targets 15, 785–788. 10.1517/14728222.2011.577420 21476883

[B95] Weaver B. A. (2014). How Taxol/Paclitaxel Kills Cancer Cells. MBoC 25, 2677–2681. 10.1091/mbc.E14-04-0916 25213191 PMC4161504

[B96] Weyburne E. S. Wilkins O. M. Sha Z. Williams D. A. Pletnev A. A. de Bruin G. (2017). Inhibition of the Proteasome β2 Site Sensitizes Triple-Negative Breast Cancer Cells to β5 Inhibitors and Suppresses Nrf1 Activation. Cell Chem. Biol. 24, 218–230. 10.1016/j.chembiol.2016.12.016 28132893 PMC5341617

[B97] Wheeler L. W. Lents N. H. Baldassare J. J. (2008). Cyclin A-CDK Activity during G1 Phase Impairs MCM Chromatin Loading and Inhibits DNA Synthesis in Mammalian Cells. Cell Cycle 7, 2179–2188. 10.4161/cc.7.14.6270 18635963

[B98] Wirth K. G. Ricci R. Giménez-Abián J. F. Taghybeeglu S. Kudo N. R. Jochum W. (2004). Loss of the Anaphase-Promoting Complex in Quiescent Cells Causes Unscheduled Hepatocyte Proliferation. Genes Dev. 18, 88–98. 10.1101/gad.285404 14724179 PMC314282

[B99] Wohlschlegel J. A. Dwyer B. T. Dhar S. K. Cvetic C. Walter J. C. Dutta A. (2000). Inhibition of Eukaryotic DNA Replication by Geminin Binding to Cdt1. Science 290, 2309–2312. 10.1126/science.290.5500.2309 11125146

[B100] Yuan X. Srividhya J. De Luca T. Lee J.-h. E. Pomerening J. R. (2014). Uncovering the Role of APC-Cdh1 in Generating the Dynamics of S-phase Onset. MBoC 25, 441–456. 10.1091/mbc.E13-08-0480 24356446 PMC3923637

[B101] Zasadil L. M. Andersen K. A. Yeum D. Rocque G. B. Wilke L. G. Tevaarwerk A. J. (2014). Cytotoxicity of Paclitaxel in Breast Cancer Is Due to Chromosome Missegregation on Multipolar Spindles. Sci. Transl. Med. 6, 229ra43. 10.1126/scitranslmed.3007965 PMC4176609 24670687

[B102] Zeidan A. M. Ridinger M. Lin T. L. Becker P. S. Schiller G. J. Patel P. A. (2020). A Phase Ib Study of Onvansertib, a Novel Oral PLK1 Inhibitor, in Combination Therapy for Patients with Relapsed or Refractory Acute Myeloid Leukemia. Clin. Cancer Res. 26, 6132–6140. 10.1158/1078-0432.CCR-20-2586 32998961

[B103] Zeng X. Sigoillot F. Gaur S. Choi S. Pfaff K. L. Oh D.-C. (2010). Pharmacologic Inhibition of the Anaphase-Promoting Complex Induces a Spindle Checkpoint-dependent Mitotic Arrest in the Absence of Spindle Damage. Cancer Cell 18, 382–395. 10.1016/j.ccr.2010.08.010 20951947 PMC2957475

[B104] Zeng X. Xu W. K. Lok T. M. Ma H. T. Poon R. Y. C. (2019). Imbalance of the Spindle-Assembly Checkpoint Promotes Spindle Poison-Mediated Cytotoxicity with Distinct Kinetics. Cell Death Dis. 10, 1–15. 10.1038/s41419-019-1539-8 PMC6450912 30952840

[B105] Zhang D. Yin S. Jiang M.-X. Ma W. Hou Y. Liang C.-G. (2007). Cytoplasmic Dynein Participates in Meiotic Checkpoint Inactivation in Mouse Oocytes by Transporting Cytoplasmic Mitotic Arrest-Deficient (Mad) Proteins from Kinetochores to Spindle Poles. Reproduction 133, 685–695. 10.1530/rep.1.01167 17504913

[B106] Zhang J. Wan L. Dai X. Sun Y. Wei W. (2014). Functional Characterization of Anaphase Promoting Complex/Cyclosome (APC/C) E3 Ubiquitin Ligases in Tumorigenesis. Biochimica Biophysica Acta (BBA) - Rev. Cancer 1845, 277–293. 10.1016/j.bbcan.2014.02.001 PMC3995847 24569229

[B107] Zhang Y. Li J. Yi K. Feng J. Cong Z. Wang Z. (2019). Elevated Signature of a Gene Module Coexpressed with CDC20 Marks Genomic Instability in Glioma. Proc. Natl. Acad. Sci. U.S.A. 116, 6975–6984. 10.1073/pnas.1814060116 30877245 PMC6452696

[B108] Zhu Y. Zhou Y. Shi J. (2014). Post-slippage Multinucleation Renders Cytotoxic Variation in Anti-mitotic Drugs that Target the Microtubules or Mitotic Spindle. Cell Cycle 13, 1756–1764. 10.4161/cc.28672 24694730 PMC4111722

